# Human resources requirements for highly active antiretroviral therapy scale-up in Malawi

**DOI:** 10.1186/1472-6963-7-208

**Published:** 2007-12-19

**Authors:** Adamson S Muula, John Chipeta, Seter Siziya, Emmanuel Rudatsikira, Ronald H Mataya, Edward Kataika

**Affiliations:** 1 Department of Community Health, University of Malawi, College of Medicine, Blantyre, Malawi; 2 Department of Epidemiology, University of North Carolina at Chapel Hill, North Carolina, USA; 3 National AIDS Commission, Lilongwe, Malawi; 4 Department of Community Medicine, University of Zambia Medical School, Lusaka, Zambia; 5 Departments of Global Health, Epidemiology and Biostatistics, School of Public Health, Loma Linda University, California, USA; 6 Department of Global Health, Loma Linda University, School of Public Health, Loma Linda, California, USA; 7 Department of Planning and Policy Development, Ministry of Health, Lilongwe, Malawi

## Abstract

**Background:**

Twelve percent of the adult population in Malawi is estimated to be HIV infected. About 15% to 20% of these are in need of life saving antiretroviral therapy. The country has a public sector-led antiretroviral treatment program both in the private and public health sectors. Estimation of the clinical human resources needs is required to inform the planning and distribution of health professionals.

**Methods:**

We obtained data on the total number of patients on highly active antiretroviral treatment program from the Malawi National AIDS Commission and Ministry of Health, HIV Unit, and the number of registered health professionals from the relevant regulatory bodies. We also estimated number of health professionals required to deliver highly active antiretroviral therapy (HAART) using estimates of human resources from the literature. We also obtained data from the Ministry of Health on the actual number of nurses, clinical officers and medical doctors providing services in HAART clinics. We then made comparisons between the human resources situation on the ground and the theoretical estimates based on explicit assumptions.

**Results:**

There were 610 clinicians (396 clinical officers and 214 physicians), 44 pharmacists and 98 pharmacy technicians and 7264 nurses registered in Malawi. At the end of March 2007 there were 85 clinical officer and physician full-time equivalents (FTEs) and 91 nurse FTEs providing HAART to 95,674 patients. The human resources used for the delivery of HAART comprised 13.9% of all clinical officers and physicians and 1.1% of all nurses. Using the estimated numbers of health professionals from the literature required 15.7–31.4% of all physicians and clinical officers, 66.5–199.3% of all pharmacists and pharmacy technicians and 2.6 to 9.2% of all the available nurses. To provide HAART to all the 170,000 HIV infected persons estimated as clinically eligible would require 4.7% to 16.4% of the total number of nurses, 118.1% to 354.2% of all the available pharmacists and pharmacy technicians and 27.9% to 55.7% of all clinical officers and physicians. The actual number of health professionals working in the delivery of HAART in the clinics represented 44% to 88.8% (for clinical officers and medical doctors) and 13.6% and 47.6% (for nurses), of what would have been needed based on the literature estimation.

**Conclusion:**

HAART provision is a labour intensive exercise. Although these data are insufficient to determine whether HAART scale-up has resulted in the weakening or strengthening of the health systems in Malawi, the human resources requirements for HAART scale-up are significant. Malawi is using far less human resources than would be estimated based on the literature from other settings. The impact of HAART scale-up on the overall delivery of health services should be assessed.

## Background

Malawi is among the countries in southern Africa with a high HIV and AIDS burden. The Malawi Demographic and Health Survey (DHS) conducted in 2004 estimated that about 12 percent of the adult population (15 years to 49 years) is infected with HIV [1]. Maternal mortality is estimated at about 1,000 deaths per 100,000 deliveries [1]. The increasing maternal mortality ratio is directly and indirectly related to HIV; directly due to a rise in maternal infections and other complications related to HIV and indirectly due to reduction of human resources as a result of AIDS-related illness and deaths among health professionals [2-4]. When the World Health Organization initiated the "3 by 5" initiative aimed to treat 3 million HIV-infected individuals with highly active antiretroviral drugs by 2005 [5,6], there was a concern about the potential negative impact of the scaling-up of Highly active antiretroviral therapy (HAART) through vertical programs on the public health system in the affected countries [7-9].

Malawi is one of the resource-limited countries that have shown commitment to provide HAART for free to all its citizens with both donor and local financial support [10-12]. Drugs are also available in the private sector for a nominal fee of about US$ 3 per month's supply. The initial stages of the program, scaling-up and lessons learnt from this HAART scaling-up have been described elsewhere [10-13]. As at the end of March 2007, a total of 95,674 individuals were on HAART [13]; 14.4% of those were enrolled in the first quarter of 2007. A total of 144 health facilities: 38 private, 26 mission; 10 NGOs; 6 Army/Police; 2 sugar plantation clinics and 62 public (owned by the Ministry of Health) were providing HAART. The public sector provides 60% of health services, the Christian Health Association of Malawi (CHAM) 37%, the Ministry of Local Government 2% while private clinics, companies and the uniformed forces provide the remaining 1% [14].

The public health-sector led antiretroviral treatment program in Malawi was founded on the following health professionals' cadres: clinical officers, physicians and nurses. Clinical officers and physicians provide clinical assessment to determine eligibility of patients to start treatment. They are also responsible for initial and follow-up prescribing. Nurses provide assessment of patients during follow-up visit, refill patients' medications without having them see a clinical officer or physician. They dispense medications and provide initial counseling and drug adherence counseling. Lay counselors support nurses in the provision of HIV pre-testing and post-test counseling.

Estimating human resources for health (HRH) requirements and implications of HAART scaling up will inform HRH planning and distribution. We therefore set out to estimate the human resources requirements for HAART scale-up in Malawi based on the national HIV burden and the available health professionals to provide care.

## Methods

We obtained HIV prevalence estimates for 2006 from the National AIDS Commission and UNAIDS [15]. We compared these to the estimated the number of HIV infected adults based on the Malawi Demographic and Health Survey and the national population [1,16-18].

We also estimated the human resource requirements for the provision of HAART to the estimated number of HIV patients eligible for HAART. We used the human resource estimations reported by Hirschhorn et al [7] which indicate that the number of health care professionals required to provide HAART to 1000 patients in Africa would be 1–2 physicians or clinical officers, 2–7 nurses, and <1 to 3 pharmacy staff [19].

Data on the numbers of physicians and clinical officers, nurses, pharmacists and pharmacy technicians were obtained from their respective regulatory or registering boards, namely the Medical Council of Malawi (clinical officers and physicians), Nurses and Midwives Council of Malawi (nurses) and Medicine and Poisons Board (pharmacists and pharmacy technicians). We also obtained data on the estimated number of nurses in Malawi from the World Health Organisation [20] for comparison with national data. Data on the number of health professionals within the Ministry of Health and CHAM health facilities and the vacancy rates were obtained from the Department of Planning and Policy Development of the Malawi Ministry of Health.

We calculated the number of health professionals that would be required to provide care to 95,674 patients on HAART using the following formula:

(α*B)/k

Where α = the number of health professionals required for 1,000 patients as estimated by Hirchhorn et al [19],

*B *= total number of patients on HAART

*K *= 1,000 patients

Using the number of health professionals within a professional category, we calculated what proportion of the total health professional pool the estimated number of health professionals required for the delivery of HAART in Malawi was.

We also used data from Van Damme *et al.*[21] to estimate need of clinicians for 95,674 and 170,000 patients. The estimated number of HIV infected persons needing HAART in Malawi in 2003 [18] was 170,000. We are unaware of any revised estimate to this 2003 figure, so we maintained the figure as a reasonable current estimate. Van Damme et al estimated in Cambodia that 2.06 FTE (full-time equivalents of doctors) would be required at the inception of a treatment program for 522 patients. In subsequent years, this would change to 1.97 FTE for 911 patients. The reduction in FTE of doctors after the first year of the program was due to the fact that doctors had gained experience and patients had been stabilized on treatment; thus resulting in a reduced patient-clinician consultation time. These estimates were then compared to the actual number of health professionals providing HAART as at end March 2007.

## Results

Based on UNAIDS reports, the estimated number of HIV infected individuals stratified by age groups in Malawi in 2006 is shown in Table 1. The number of adults infected with HIV in Malawi was 940,000 (range 440,000–1,300,000) in 2006. Further description of numbers of infected person per age groups is shown in Table 1. While the UNAIDS estimate for the group 15 to 49 years was 850, 000, based on the HIV prevalence from Demographic and Health Survey (11.8%) and the population projections from the National Statistical Offices (4,397,545), the number of HIV infected individuals in this age group would be 518,910 i.e. 55.2% of the UNAIDS estimate (range 37.1% to 108.1%).

**Table 1:** HIV burden in Malawi 2006

**Demographic category**	**Number Infected**	**Lower and Upper bounds**
Total number of people infected	940,000	480,000–1,400,000
Adults 15 to 49 years infected	850,000	440,000–1,300,000
Children 0 to 14 years infected	91,000	28,000–91,000

There were 214 physicians registered with the Medical Council of Malawi in 2006. Three physicians registered on the temporary register were excluded as it was perceived these were not likely to be in the pool that would be mobilized for HAART delivery. The number of clinical officers was 396, thus the total number of clinicians (medical doctors and clinical officers) was 610. There were 46 pharmacists and 98 pharmacy technicians registered with the Pharmacy, Medicine and Poison Board of Malawi in March 2007.

The World Health Organization (WHO) estimated that there were 7264 nurses in Malawi in 2006 [20]. However, the number reported as registered by the Nurses and Midwives Council of Malawi as at end 2006 was 4211 distributed as 2800 enrolled nurse-midwives, 635 nurse-midwife technicians, 533 registered nurses (diploma and degree nurses) and 145 nurse-technicians. The proportions of health professionals available in established posts and the vacancies reported by the Ministry of Health (for both the Ministry of Health and CHAM) are shown in Table 2. However Table 2 only reports those health workers employed by the Ministry of Health and CHAM; thus excluding the private sector whose requirements are not known. As shown in Table 2, CHAM and Ministry of Health nurses were 4717 in 2005.

**Table 2:** Required Human Resources for Government and CHAM Health Facilities, 2005

Cadre	Ministry of Health and CHAM Target Cadre	Current Staff	Current Vacancies	% Actual Staff in Post to Target	% Vacant Posts
Physicians	433	139	294	32	68
Nurses	8,440	4,717	3,723	56	44
Clinical Officers	1,405	942	463	67	33
Medical Assistants	1,500	718	782	48	52
Lab Technicians	507	251	256	50	50
Pharmacists	285	93	192	50	50
Environmental Health Officers	1,662	304	1,358	18	82

The number of nurses reported by the Nurses and Midwives Council of Malawi was probably an under-estimate, as it is even lower than the actual numbers of nurses employed within the Ministry of Health and CHAM. It is for this reason that our subsequent analyses will assume that the WHO figure is more realistic. In any case however, the vacancy rates for all health professional cadres reported range from 33% to 82%.

Using Hirschhorn *et al.*[19] formula, the number of health professionals required to deliver HAART to 95,674 patients is shown in Table 3. The largest proportional burden on a health worker category is on pharmacists and pharmacy technicians (Table 3). In this category, more than half of the health professionals (66.5%–199.3%) would be required.

**Table 3:** Estimated human resources requirements for 95,674 HAART patients in Malawi, 2007

**Health professional category**	**Number registered**	**Number of workers required for HAART provision**	**Expressed as % of total health professionals in cadre**
Physicians and clinical officers	610	95.7–191.3	15.7–31.4
Pharmacists and technicians	144	95.7–287.0	66.5–199.3
Nurses	7264	191.3–669.7	2.6–9.2

The Malawi National AIDS Commission (NAC) estimated in 2004 that about 170,000 individuals were clinically eligible for HAART [10]. We therefore estimated how many clinicians, nurses and pharmacists and pharmacy technicians would be required to deliver HAART to those patients. In order to provide care to all the 170,000 HIV infected persons estimated to be clinically eligible for HAART, it would require 4.7% to16.4% of the total number of nurses, 118.1% to 354.2% of all available pharmacists and pharmacy technicians and 27.9% to 55.7% of all clinical officers and physicians.

Based on Van Damme et al's physician requirements for the provision of HAART (2.06 FTE per 1,000) [20], we estimated that taking care of 13,770 who had recently initiated treatment, would require 28.3 FTE of clinical officers and physicians. The remaining 81,904 patients assumed to have been stabilized, at 1.97 FTE per 1,000 patients, would require 161.4 FTE. The total requirements would be 189.7 FTE. As a proportion of the available clinical officers and physicians, these human resources requirements would translate to 31.1% of all clinicians.

We also obtained data from the Malawi Ministry of Health on how many health professionals were actually providing HAART care. Table 4 shows the number of health professional days per week for physicians and clinical officers and nurses. The FTE parameters indicate the number of clinical officers and physicians and nurses working full-time per week on ART. Thus, for the whole country, the equivalent of 85 FTE (physicians and clinical officers) and 91 FTE (nurses) were working full-time in ART delivery each week. Estimates for pharmacists and pharmacy technicians were not available. Comparing these actual FTEs to the available pool of health professionals indicates that 13.9% of all clinical officers and physicians and 1.1% of nurses would have to work full-time providing HAART in Malawi for the current number of patients on treatment (95, 674). If however instead of using both clinical officers and physicians only physicians are considered, the need would be 92.9% of the 214 physicians in the country.

**Table 4:** Actual numbers of health professionals delivering HAART in Malawi, 2007

Region of the country	Number of sites proving HAART	Physicians and clinical officers' days/week	Nurse days/week
North: 22 sites	22	55	60
Central: 39 sites	39	161	174
South: 45 sites	45	211	223
National	106	427	457
FTEs	176	85	91

The comparison between findings reported in Tables 3 and 4 indicates that Malawi is using fewer human resources than would be expected or experienced in other settings. For the 95,674 patients, between 95.7 FTEs and 191.3 FTEs for clinical officers and medical doctors' would be needed. Furthermore, nurses have taken on the role of dispensing medications within the program such that the contribution of pharmacists and pharmacy technicians in insignificant. The Malawi program was using 85 FTEs for all its patients. With regard to nurses, 191.3 to 669.7 FTEs would be needed but only 91 FTEs were being used. The actual number of health professional working in the delivery of HAART in the clinics represented 44.0% to 88.8% (for clinical officers and medical doctors) and 13.6% and 47.6% (for nurses), of what would have been needed based on van Damme and Hisschhorn [19,21].

## Discussion

We have reported the estimated human resources needs for HAART scale-up in Malawi. We report that 13.9% of FTEs of all clinical officers and physicians and 1.1% of nurse would currently be devoted to providing HAART in Malawi for the 95, 674 patients on treatment as of March 31, 2007. However based on experiences from elsewhere [19], at least 16.7% of clinical officers and physicians, 66.5% to 199% of pharmacists and pharmacy technicians and 2.6% to 9.2% of nurses would be required. Using a physician-oriented treatment program as has been reported by van Damme [21], Malawi would need to devote 31.1% of all its clinical officers and physicians working full-time to provide HAART. However, if the treatment programs were only to consider physicians, thus excluding clinical officers, almost all (92.9%) of the available physicians would have to work full time in the HAART delivery program.

The estimation that 1 to 2 clinicians as estimated by Hirschorn [19] may be required for 1,000 patients may not be an exaggeration. There are 5 working days in a week and for a 30 day month, a clinician may have to see not more than 45 to 50 patients each day to reach a 1,000 patients target. In any case also, considering breaks and weekends, the work-week may in fact be less than 5 working days. Being consulted by 45 to 50 HAART patients each day is a huge workload, but necessary if HAART scale-up is to be maintained.

As shown in Table 4, the actual numbers of health professionals used in the Malawi HAART program is much lower than would be expected using estimates from van Damme [19] and Hirschhorn [21]. This would suggest that programs elsewhere may be even more labor intensive than the Malawi program. So far, published data has not shown compromised patient drug adherence in Malawi [22] as compared to other settings. This however needs to be evaluated further.

The limited number of health professionals available in Malawi has resulted from multiple factors. These include migration, especially of nurses, to developed nations [23,24], limited number of all cadres graduating from training institutions and lack of training programs for pharmacists [25,26]. Between 2000 and 2005, the Nurses and Midwives Council of Malawi validated 616 nurses for practice in other countries [27]. Until 2005, Malawi was only able to train pharmacy assistants and technicians. A Bachelor of Science Pharmacy training program was initiated in 2006 at the Malawi College of Medicine with some financial support from the Global Fund.

Although there continues to be limited numbers of health workers in Malawi, most training institutions have increased their enrollments, and in some cases more than doubled the enrolment compared to enrollments before 2000. The College of Medicine which is the only medical school in Malawi originally had an annual intake of between 14 to 20 students, graduating on average 17 students each year [28]. Currently, there is an annual intake of 50 to 60 students.

A study conducted in six selected districts in 2004 reported the following major causes of health professionals' attrition: death (48%), resignations (38%), and early retirement (9%) [29]. At least 68 percent of the workforce left when they had served between two and 10 years of service [28].

Record and Mohiddin [30] have argued that the migration of health workers from Malawi may be beneficial to the country in part due to the remittances that the country may stand to gain. With the current human resources limitations the country is facing, we argue that no financial gain could be justified at the expense of health services delivery. However, the reasons for the migration of health professions must be addressed to ensure human resources retention in Malawi.

The human resources challenges faced by Malawi are not insurmountable. Harries *et al.*[31] have suggested several solutions which include training, retention and motivation, occupational health services including access to HAART and proper planning and management of human resources.

The Malawi Ministry of Health's HIV Unit has come up with an initiative of rewarding good performance in ARV clinics. This is done by issuing quarterly certificates for excellent performance. The certificate is signed by the Secretary for Health and is displayed for public information. This is a popular and an inexpensive way to motivate staff. From October 2006 to July 2007, between 50% and 60% of the HIV treatment sites have received certificates of excellence in each of the quarters.

The HIV epidemic in Malawi has affected the available number of health professionals through deaths, absenteeism and retirement on medical grounds [13,32]. However HAART has the potential to reduce some of these losses as many of the affected individuals may return to work. As at end of March 2007, there were 1443 healthcare workers on HAART, thus comprising about 2% of all patients receiving antiretroviral treatment. Of these, 158 had started treatment in the first quarter of 2007 [13].

The Malawi Ministry of Health has taken an initiative called "task shifting", which implies "shifting tasks among and between cadres of health workers and to trained members of the community including people living with HIV." This has potential to rapidly expand the resource pool and to maximize the availability of the more skilled workers [33]. Starting in the second quarter of 2007, the Ministry of Health's HIV Unit involves nurses and medical assistants training in HAART clinical management of HIV patients to prescribe treatment. Patients are only seen monthly for the first six months of treatment. If patients have stabilized, they only see nurses at these visits. After six months of therapy, if patient continues to be stable, they are provided two monthly drug supplies (compared to monthly supplies prior to this time). This task shifting will certainly enable the Ministry of Health to increase the number of patients on HAART since the nurse pool, although overstretched with other responsibilities, remains the largest group of health professionals from which clinical staff could be drawn.

The Malawi's HAART program could not expand to over 100,000 patients if it solely relied on medical doctors and clinical officers for prescribing authority. There is evidence both in Malawi and elsewhere that non-physician health professionals can provide selected clinical and surgical care comparable to physicians [34-36]. There is also need to explore whether health surveillance assistants, lay counselors and expert patients could be enlisted to provide services such as patients' adherence counseling and other responsibilities. HIV pre-test and post-test counseling in Malawi is largely provided by lay counselors. Also there is need to explore possibilities of nurse-prescribers in Malawi not only for HAART but for other conditions.

The original design of the Malawi HAART program which preferred use of clinical officers and physicians for prescribing authority favored urban areas where these cadres, although few in number, are concentrated. In general, rural health centers are staffed with nurses and medical assistants. The shift in policy to include nurses and medical assistants will potentially increase the access to HAART by patients in rural areas. The quarterly supervisory capacity of the Ministry of Health's HIV unit will also be challenged with increased numbers of clinics in rural remote areas.

One of the benefits of the HAART program with regard to human resources is that the availability of HAART prevents occurrence of opportunistic infection (OIs). When OIs are prevented patients may not need as many hospital visits [37].

Laleman et al [38] have reported that international health volunteers contribute relatively small numbers to the health workforce in sub-Saharan Africa. This may certainly be the case at the continental level, but the situation may be viewed differently for Malawi. For instance, a country like Malawi which has one radiologist, three pathologists, and small numbers of health professionals, the services of even a handful of volunteer health workers make a difference.

The Malawi HAART program uses far less number of health professionals that would have been expected based on the estimates from other settings. This could be a model to other countries in Southern Africa with a high HIV burden but low human resources pool to draw from. There is also need to explore whether the changes in policies (task shifting and reduced number of visits) have not compromised the quality of care. We would only know if this is assessed in the future.

Our assessment of the potential consequences of HAART scale-up is not intended to suggest that HIV-infected persons do not deserve care and the associated human resources commitment. Our motivation is to demonstrate the impact one disease (HIV/AIDS) has had on human resources on an already fragile health system.

## Limitations of the study

Our study has a number of limitations. Not all health professionals registered in Malawi are within the country. Some health professionals, such as those in administrative positions, may be registered by their respective regulatory bodies but unavailable to provide patient care. This would suggest that the pool available for the delivery of care is less than what we have reported. It is also possible that some health professionals may not be registered with the respective regulatory bodies. We believe that the number of such non-registered health professionals is minimal. The Medical Council of Malawi levies a 50 percent penalty on annual registration fees for a health professional who has not renewed their registration past 30 days at the expiration of the financial year. Such penalty is a strong disincentive against practicing while not registered.

Although we have used Hirschhorn et al's estimate [19] to calculate Malawi's estimated human resources needs, the actual requirements may be different as patients initiating care and those that have been stabilized require different amounts of clinic time resources [21]. Van Damme *et al.*[21] estimated that on average, doctors in Cambodia were seeing patients for about 10 minutes at each visit. Reducing the patient-clinician consultation time may reduce the overall number of clinician caring for patients on HAART, but may also compromise the quality of care that patients receive.

While the Malawi HAART program has been clinical officer and nurse driven, with a smaller contribution by physicians, the estimates provided from van Damme *et al.*[21] are based on a solely physician led program. The conditions in those programs may not be transferable to the Malawi program.

When the actual numbers of health professionals currently providing care are compared to the theoretical estimates obtained from Hirschhorn and van Damme, we found that a smaller proportion of physicians and clinical officers were actually delivering care. Our study did not assess the quality of care offered to patients based on waiting times, adequacy of time clinician spends with patients, patient advice and assessment. We are therefore unable to determine whether the lower number of health professionals used in Malawi compared to the theoretical estimates has led to compromise in the care provided. As the scaling up of HAART continues, there is need to assess the quality of care and identify ways care could be improved.

## Conclusion

HAART provision is a labour intensive exercise. Although these data are insufficient to determine whether HAART scale-up has resulted in the weakening or strengthening of the health systems in Malawi, the human resources requirements for HAART scale-up are significant. Malawi is using far less human resources than would be estimated based on the literature from other settings. The scaling up of HAART delivery in Malawi will demand scaling-up in human resources planning, training, retention and management. There is also need for an audit to assess the impact of HAART on other health services delivery. Given the similarities between Malawi and other countries in the region, findings from this study have implications in other countries in Sub-Saharan Africa. Realization of the G8's declaration "Universal Access to ART" by 2010 [39] will only be achieved if there is a massive investment in training and retention of health professionals in Africa.

## List of abbreviations

AIDS: acquired immunodeficiency syndrome

ART: antiretroviral therapy

DHS: Demographic and Health Survey

HAART: highly antiretroviral therapy

HIV: human immunodeficiency virus

FTE: full-time equivalents

NAC: National AIDS Commission

WHO: World Health Organisation

## Competing interests

The author(s) declare that they have no competing interests.

## Authors' contributions

ASM conceived the study, conducted data analysis and participated in drafting manuscript.

JC: Collected data and participated in the interpretation of the findings.

SS: participated in the interpretation and drafting of the manuscript.

ER: participated in the interpretation and drafting of manuscript.

RMH: participated in the data collection, interpretation of the findings and drafting of manuscript.

EK: participated in data collection and drafting of manuscript.

## Pre-publication history

The pre-publication history for this paper can be accessed here:


